# Association between napping and 24-hour blood pressure variability among university students: A pilot study

**DOI:** 10.3389/fped.2023.1062300

**Published:** 2023-03-02

**Authors:** Jie Dai, Hua-ying Wu, Xiao-dong Jiang, Yong-jie Tang, Hao-Kai Tang, Li Meng, Na Huang, Jing-yu Gao, Jian Li, Julien S. Baker, Chan-Juan Zheng, Yi-De Yang

**Affiliations:** ^1^ Department of Child and Adolescent Health, School of Medicine, Hunan Normal University, Changsha, China; ^2^ Key Laboratory of Molecular Epidemiology of Hunan Province, School of Medicine, Hunan Normal University, Changsha, China; ^3^ Key Laboratory of Study and Discovery of Small Targeted Molecules of Hunan Province, School of Medicine, Hunan Normal University, Changsha, China; ^4^ Centre for Health and Exercise Science Research, Department of Sport, Physical Education and Health, Hong Kong Baptist University, Hong Kong, Hong Kong SAR, China

**Keywords:** napping duration, blood pressure variability, 24-h ambulatory blood pressure monitoring, university students, average real variability

## Abstract

**Background:**

Blood pressure variability (BPV) has been reported to be a predictor of cardiovascular and some cognitive diseases. However, the association between napping and BPV remains unknown. This study aimed to explore the association between napping and BPV.

**Materials and methods:**

A cross-sectional study including 105 university students was conducted. Participants’ 24 h ambulatory blood pressure monitoring (24 h ABPM) were measured, and napping behaviors were investigated. BPV were measured by the 24 h ABPM, included standard deviation (SD), coefficient of variation (CV), and average real variability (ARV).

**Results:**

Among the participants, 61.9% reported daytime napping. We found that nap duration was significantly associated with daytime CV of diastolic blood pressure (DBP) (*r* = 0.250, *P *= 0.010), nighttime CV of systolic blood pressure (SBP) (*r* = 0.217, *P *= 0.026), 24 h WCV of DBP (*r* = 0.238, *P *= 0.014), 24 h ARV of SBP (*r* = 0.246, *P *= 0.011) and 24 h ARV of DBP (*r* = 0.291, *P *= 0.003). Compared with the no napping group, 24 h WCV of DBP, daytime CV of DBP, and daytime SD of DBP were significantly higher in participants with napping duration >60 min. With multiple regression analysis we found that nap duration was an independent predictor for 24 h ARV of SBP (*β* = 0.859, 95% CI, 0.101–1.616, *P *= 0.027) and 24 h ARV of DBP (*β* = 0.674, 95% CI, 0.173–1.175, *P *= 0.009).

**Conclusions:**

Napping durations are associated with BPV among university students. Especially those with napping durations >60 min had a significantly higher BPV than those non-nappers.

## Introduction

Blood pressure variability (BPV) is a concept used to characterize continuous dynamic fluctuations in blood pressure. BPV can be described in short-term variability (within a day, 24 h BPV) and long-term variability (between clinic visits over months and years, visit-to-visit BPV) (1). There are some common indicators of BPV, including standard deviation (SD), range, coefficient of variation (CV), and average real variability (ARV) (2). BPV has been reported to be risk a factor or associated with the progression of many diseases (3–7). Among patients with Parkinson's disease(PD), 24 h CV of DBP in the advanced PD group was significantly higher than that in the control group and the early PD group (3). Also, BPV was found to be related to the severity of obstructive sleep apnea (OSA) (4). Laure et al. showed that higher systolic BPV was associated with higher risk of dementia (HR = 1.23, 95% CI, 1.01–1.50) and it could be a major clinical predictor of cognitive impairment and dementia (5). Most importantly, sustained increases in BPV may reflect alterations in cardiovascular regulatory mechanisms or underlying pathological conditions (6). Accumulating evidence suggests that sustained increase in BPV is associated with an increased risk of subclinical organ damage, cardiovascular events, and all-cause mortality (7).

Among university students, late bedtime and daytime napping are extremely prevalent, due to a variety of factors including academic stress, socialization, coffee intake, and uncontrolled use of social media (8). A meta-analysis of sleep duration and sleep patterns among Chinese university students showed that the proportion of students who slept less than 6 h/day and 7 h/day (short sleep duration) was 8.4% and 43.9%, respectively, and the mean bedtime was 00:51 AM (9). Short sleep duration at night may contribute to a substantial increase in the duration and frequency of daytime napping which might also impact the incidence of cardiovascular diseases (10, 11). Previous studies reported a 2.20-fold increase in the risk of heart failure in participants who napped <1.7 times per day compared to those who napped >1.7 times per day (12). In another cohort study of older adults, the risk of hypertension was found to be 1.18 times higher in those with longer nap durations (≥90 min) than in non-nappers (13). As we all know, the 24 h sleep-wake cycle is closely related to 24 h blood pressure fluctuations (14, 15). Longer daytime napping was found to be linked with lower sleep quality in a global survey of athletes (16). Participants with low sleep quality (inefficient sleep) have been reported to a high prevalence of elevated short-term BPV (17). However, there is little evidence available that relates to the relationship between napping duration and BPV. In the present study, we hypothesized that napping duration potentially leads to abnormal fluctuations in blood pressure.

## Materials and methods

### Study population, sample size calculation and study procedures

Our study was conducted in a University in Hunan Province, China from 2020 to 2021. The study recruited university students through a convenience sampling method by flyers or posters. The sample size of the present correlation study was calculated using PASS 2021. Since no previous studies have reported the association between nap and BPV. We used the correlation coefficients between sleep and blood pressure in previous studies, previous studies reported correlations within the range of between 0.25 to 0.45 (18, 19). Determination of the minimum sample size was calculated by the following estimates: (i) expected correlation between the two variables (*r* = 0.35), (ii) statistical power = 90%, (iii) Alpha = 5%, (iv) correlation coefficient of the null hypothesis (*r* = 0.0), (v) dropout rate = 20%. The sample size of 102 could provide 90% statistical power to test the significant correlations.

In our study, we included university students who (i) provided written informed consent in the study, (ii) are university students during the 2020–2021 period; (iii) students with normal sleep cycle (no medical intern with shift work involved). The exclusion criteria included the following: (i) pregnancy, (ii) taking antihypertensive and hypnotics, (iii) hyperthyroidism, (iv) sleep disturbance or drinking caffeine on the day of the trial, (v) incomplete sleep records, (vi) 24 h ambulatory blood pressure monitoring was ineffective. General characteristics (including age, nation, sex, household income, birth, smoking, drinking, etc) were measured by questionnaire. Moreover, all participants underwent physical examinations (including weight, height, and blood pressure).They completed the 24 h ambulatory blood pressure monitoring (24 h ABPM). Weight was measured using calibrated body composition apparatus (TANITA-MC780MA) and height was measured using a stadiometer. The present study was approved by the Research Ethical Review Committee Board of Hunan Normal University (2019–88). Finally, 105 students were included in the final analysis.

### Measurements

#### 24 h ABPM and blood pressure variability assessment

All participants were scheduled to undergo 24 h ABPM measurement using a noninvasive, validated device (Mobil-O-Graph NG, Germany). During the 24 h measurement period, the device was programmed to record readings of each parameter every 30 min during the nighttime (between 23:00 to 7:00) and every 15 min during the daytime (between 7:00–22:59). Recordings will be set to read missing values if they were outside systolic blood pressure (SBP) readings of 60–280 mmHg and diastolic blood pressure (DBP) readings of 30–190 mmHg. The recordings including at least 10 during the daytime and 5 during the nighttime were considered to be valid. (17). Blood pressure values <130/80 mmHg over 24 h, <135/85 mmHg during the daytime and <120/70 mmHg at night were categorized as normotensive (20).

Total number of measurements was summarized and the following parameters of short BPV were calculated. Basic indicators (standard deviations (SD) and coefficients of variation (CV = SD* 100/BP) for SBP and DBP) were calculated by ABPM directly. The average real variability (ARV) calculates the average of the differences (in absolute value) between consecutive BP readings: $\mathrm{ARV}=1/(N-1)\overset{N-1}{\underset{K=1}{\sum }}|BP_{k+1}-BP_{k}|$ (2). Weighted standard deviation (WSD) was calculated using the following formula: WSD = (daytime SD *number of hours awake) + (nighttime SD *number of hours asleep)/24 (21). The weighted coefficients of variation (WCV) were calculated in the similar way.

### Sleep characteristics

Throughout the 24 h ABPM period, participants were asked to fill out a 24 h behavior diary that included daytime naps and nighttime sleep. Participants were asked to fill diary sleep records at the end of the day. Based on the diary, we obtained the following indicators: nap duration, nap frequency, bedtime, time of awakening, and duration of nighttime sleep.

### Statistical analysis

All statistical analyses were performed using IBM SPSS 20.0 for Windows (SPSS Inc., Chicago, IL, United States) and R Studio software. Participants’ 24 h ABPM indicators, basic characteristics and sleep were described using mean (±SD) and frequency (%). Pearson's correlation coefficient and multiple linear regression was used for analyzing the relationship between daytime nap duration and BPV. To test the potential nonlinear associations between napping and blood pressure variability (24 h ARV, WCV and day/night CV), we used restricted cubic spline regression models with age and sex adjusted. In addition, we divided the nap duration into three groups: no napping (0 min), 0**–**60 min, and >60 min. LSD-t test was conducted for pairwise comparison between groups to detect whether there were significant differences in BPV within the groups.

## Results

### General characteristics

A total of 105 university students (20 males and 85 females) were included for the present study. Their mean age was 18.84 ± 1.21 years, mean BMI was 21.01 ± 3.05 kg/m^2^, mean bedtime was 00:19, mean nighttime sleep duration 7.52 ± 1.33 h/day, and total sleep time 8.16 ± 1.44 h/day. The proportion of students who slept no more than 6 h/day and ≤7 h/day was 8.57% and 48.57%. There were 61.9% of participants reported daytime napping and the average duration of napping was 1.05 ± 0.56 h/day.

Among the 105 university students, 68.60% had normotensive 24 h, daytime and also nighttime blood pressure. The mean 24 h SBP was 107.03 ± 6.81 mmHg, mean 24 h DBP 66.50 ± 5.32 mmHg, mean daytime SBP 108.66 ± 7.06 mmHg, mean daytime DBP 68.10 ± 5.70 mmHg, mean nighttime SBP 101.76 ± 7.21 mmHg, and mean nighttime DBP 61.83 ± 5.90 mmHg (Table 1).

**Table 1:** General characteristics of participants.

	Mean ± SD/*N* (%), (x ± s)
Age, year	18.84 ± 1.21
Male	85 (80.95%)
BMI (Kg/m^2^)	21.01 ± 3.05
24 h SBP (mmHg)	107.03 ± 6.81
24 h DBP (mmHg)	66.50 ± 5.32
Daytime SBP (mmHg)	108.66 ± 7.06
Daytime DBP (mmHg)	68.10 ± 5.70
Nighttime SBP (mmHg)	101.76 ± 7.21
Nighttime DBP (mmHg)	61.83 ± 5.90
24 h CV of SBP (%)	10.45 ± 2.18
24 h CV of DBP (%)	14.20 ± 2.68
24 h WCV of SBP	9.77 ± 2.00
24 h WCV of DBP	13.13 ± 2.56
Daytime CV of SBP (%)	9.99 ± 2.31
Daytime CV of DBP (%)	13.18 ± 3.08
Nighttime CV of SBP (%)	9.03 ± 2.48
Nighttime CV of DBP (%)	12.92 ± 3.59
24 h SD of SBP (mmHg)	11.25 ± 2.51
24 h SD of DBP (mmHg)	9.50 ± 1.72
24 h WSD of SBP	10.52 ± 2.32
24 h WSD of DBP	8.87 ± 1.79
Daytime SD of SBP (mmHg)	10.90 ± 2.71
Daytime SD of DBP (mmHg)	9.08 ± 2.17
Nighttime SD of SBP (mmHg)	9.27 ± 2.66
Nighttime SD of DBP (mmHg)	8.20 ± 2.70
ARV of SBP	9.91 ± 2.66
ARV of DBP	7.99 ± 1.87
Bedtime	00:19
Nighttime sleep duration (hours/day)	7.52 ± 1.33
Nighttime sleep duration ≦6 h (%)	9(8.57%)
Nighttime sleep duration ≦7 h (%)	57 (48.57%)
Napping (%)	65(61.90%)
Nap duration	
no napping (0 min)	40(38.10%)
0–60 min	39(37.14%)
>60 min	26 (24.76%)
Nap duration for participants with napping (hours/day)	1.05 ± 0.56
Total sleep duration (hours/day)	8.16 ± 1.44

BMI, body mass index; DBP, diastolic blood pressure; SBP, systolic blood pressure; SD, standard deviation; CV, coefficient of variation; WSD, weighted standard deviation; WCV, weighted coefficient of variation; ARV, average real variability.

### Correlation between napping and 24 h ABPM parameters

We found that nap duration was significantly correlated with daytime CV of DBP (*r* = 0.250, *P* = 0.010), nighttime CV of SBP (*r* = 0.217, *P *= 0.026), 24 h SD of DBP (*r* = 0.193, *P *= 0.036), daytime SD of DBP (*r* = 0.201, *P *= 0.040), nighttime SD of SBP (*r* = 0.222, *P *= 0.023), 24 h WCV of DBP (*r* = 0.238, *P *= 0.014), 24 h WSD of DBP (*r* = 0.202, *P *= 0.038), 24 h ARV of SBP (*r* = 0.246, *P *= 0.011) and 24 h ARV of DBP (*r* = 0.291, *P *= 0.003) (Table 2).

**Table 2:** Correlation between napping duration and 24 h ABPM parameters.

	SBP		DBP	
	*r*	*P*	*r*	*P*
24 h BP	0.086	0.386	−0.052	0.600
Daytime BP	0.052	0.596	−0.005	0.958
Nighttime BP	0.134	0.173	0.089	0.364
24 h CV	0.094	0.340	0.177	0.070
24 h WCV	0.146	0.137	0.238	0.014*
Daytime CV	0.087	0.380	0.250	0.010*
Nighttime CV	0.217	0.026*	0.056	0.572
24 h SD	0.096	0.332	0.193	0.036*
24 h WSD	0.145	0.139	0.202	0.038*
Daytime SD	0.090	0.362	0.201	0.040*
Nighttime SD	0.222	0.023*	0.074	0.450
24 h ARV	0.246	0.011*	0.291	0.003*

SD, standard deviation; CV, coefficient of variation; WSD, weighted standard deviation; WCV, weighted coefficient of variation; ARV, average real variability.

* *P *< 0.05,

### Comparison of 24 h ABPM indicators in different nap duration groups

**Figure 1:**
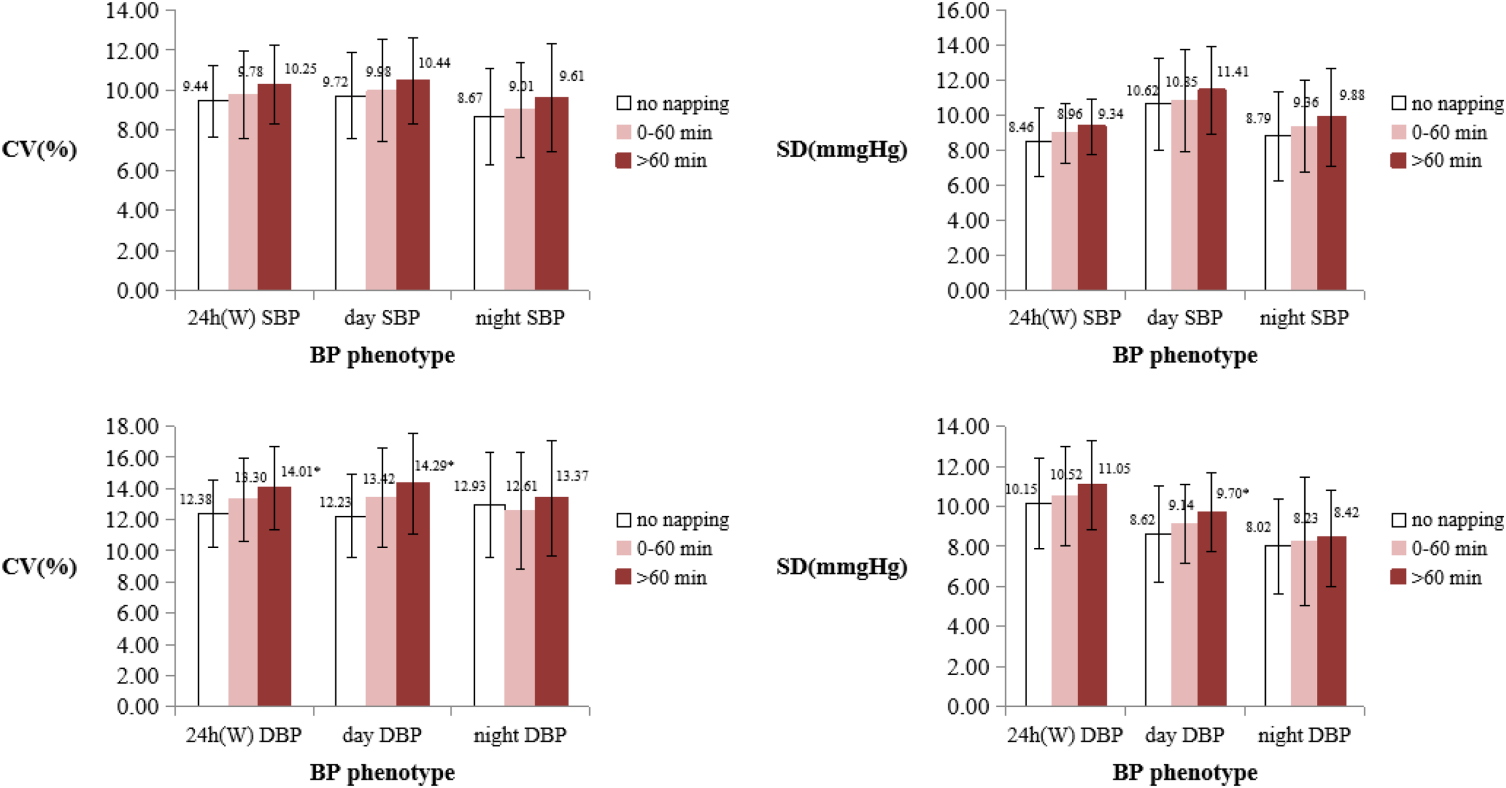
Comparison of BPV between different nap duration groups. BPV, blood pressure variability; DBP, diastolic blood pressure; SBP, systolic blood pressure; SD, standard deviation; CV, coefficient of variation; W, Means weighted. *Means that the BPV indicator is significantly different from the no napping group (*P *< 0.05).

The nap duration was categorized into three groups: no napping (0 min), 0–60 min, and >60 min. A pairwise comparison revealed that there was significant difference between the nap duration >60 min group and the no napping (0 min) group, and 24 h WCV of DBP, daytime CV of DBP, and daytime SD of DBP were higher in the nap duration >60 min group than the no napping group (*P *< 0.05) (Figure 1).

### Test for the nonlinear associations between napping and blood pressure variability indicators

**Figure 2:**
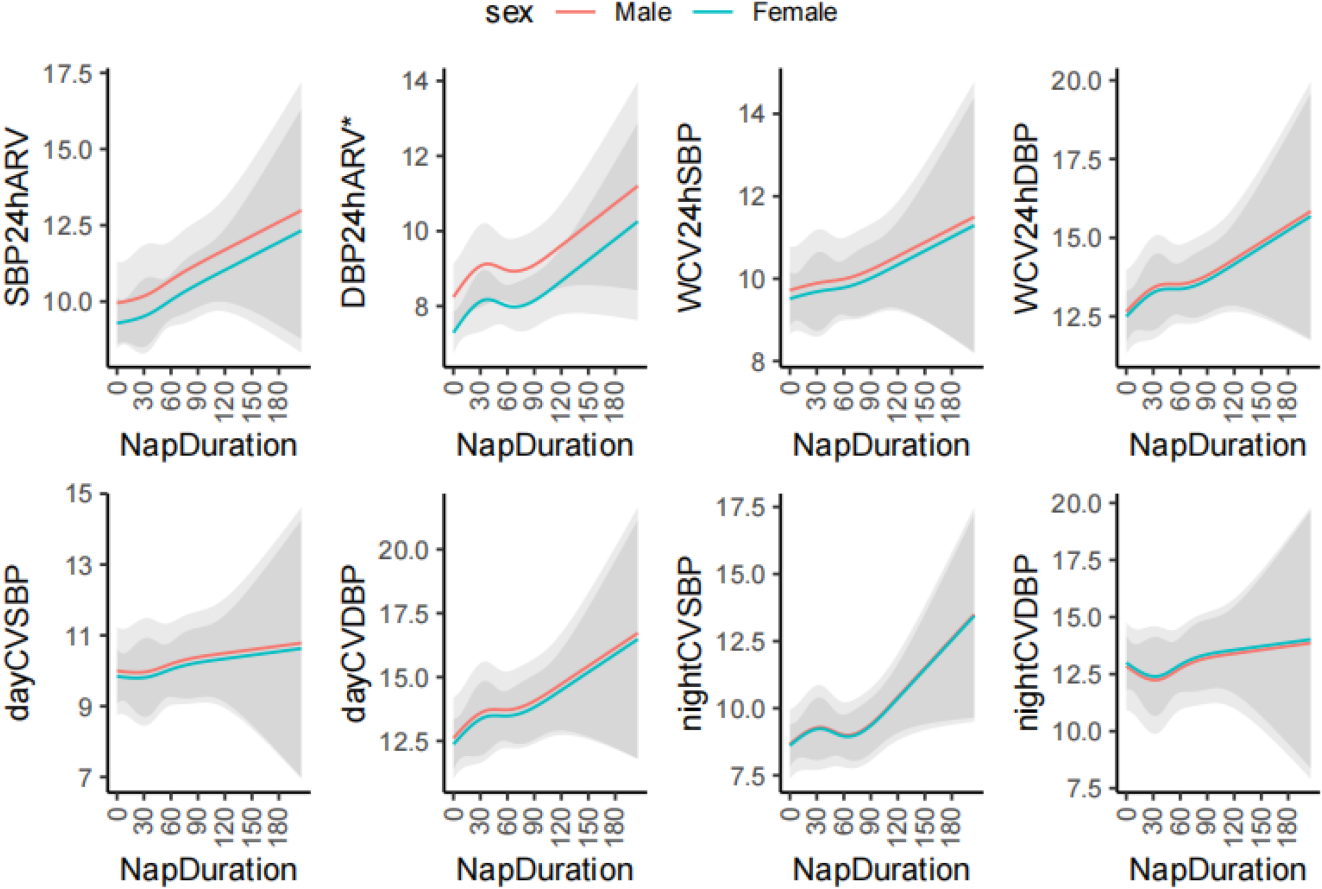
The restricted cubic spline analysis of association between nap duration and blood pressure variability. ARV, average real variability; DBP, diastolic blood pressure; SBP, systolic blood pressure; SD, standard deviation; CV, coefficient of variation; W, Means weighted. *P < 0.05. The cubic spline model was adjusted for age.

Using restricted cubic spline regression model, Figure 2 demonstrates the nonlinear correlation between napping and blood pressure variability indicators. With adjustment of age and sex, restricted cubic spline models suggested that the associations between napping and BPV (24 h ARV, WCV and day/night CV) of SBP/DBP were linear (*P* for nonlinearity = 0.990, 0.441, 0.919, 0.886, 0.958, 0.878, 0.341, and 0.859, respectively) (Figure 2).

### Multiple linear regression of BPV

Multiple linear regression analysis was performed to identify potential predictors of BPV. Nap duration was independently associated with nighttime CV of SBP and daytime CV of DBP, 24 h WCV of DBP, 24 ARV of SBP and 24 h ARV of DBP (*P *< 0.05). Longer nap duration was associated with higher daytime CV of DBP (*β* = 1.052, 95% CI, 0.167–1.937, *P *= 0.020), higher CV of SBP at night (*β* = 0.785, 95% CI, 0.062–1.509, *P *= 0.034), higher 24 h WCV of DBP (*β* = 0.827, 95% CI, 0.088–1.565, *P *= 0.029), higher 24 h ARV of SBP (*β* = 0.859, 95% CI, 0.101–1.616, *P *= 0.027) and higher 24 h ARV of DBP (*β* = 0.674, 95% CI, 0.173–1.175, *P *= 0.009) (Tables 3, 4).

**Table 3:** Multiple linear regression analysis of predictors of CV.

Outcome variables	Independent Variables	*β*	95% CI	*t*	*P*
Daytime CV of DBP	Sex	−0.202	(−1.741, 1.337)	−0.260	0.795
	Age	−0.283	(−0.790, 0.224)	−1.107	0.271
	BMI	0.097	(−0.098, 0.292)	0.987	0.326
	Nap duration (hours/day)	1.052	(0.167, 1.937)	2.359	0.020*
	Nighttime sleep duration (hours/day)	0.097	(−0.351, 0.545)	0.431	0.667
Nighttime CV of SBP	Sex	0.049	(−1.209, 1.307)	0.077	0.939
	Age	−0.153	(−0.568, 0.261)	−0.735	0.464
	BMI	0.002	(−0.157, 0.162)	0.027	0.979
	Nap duration (hours/day)	0.785	(0.062, 1.509)	2.154	0.034*
	Nighttime sleep duration (hours/day)	0.149	(−0.217, 0.515)	0.807	0.422
24 h WCV of DBP	Sex	−0.129	(−1.414, 1.155)	−0.200	0.842
	Age	−0.235	(−0.659, 0.188)	−1.102	0.273
	BMI	0.074	(−0.089, 0.237)	0.902	0.369
	Nap duration (hours/day)	0.827	(0.088, 1.565)	2.230	0.029*
	Nighttime sleep duration (hours/day)	0.017	(−0.357, 0.391)	0.091	0.928

Sex: Male = 1, Female = 2. BMI, body mass index; DBP, diastolic blood pressure; SBP, systolic blood pressure; CV, coefficient of variation; WCV, weighted coefficient of variation.

* *P *< 0.05.

**Table 4:** Multiple linear regression analysis of predictors of ARV.

Outcome variables	Independent Variables	*β*	95% CI	*t*	*P*
24 h ARV of SBP	Sex	−0.665	(−1.982, 0.652)	−1.100	0.319
	Age	−0.139	(−0.573, 0.295)	−0.635	0.527
	BMI	0.116	(−0.051, 0.283)	1.373	0.173
	Nap duration (hours/day)	0.859	(0.101, 1.616)	2.250	0.027*
	Nighttime sleep duration (hours/day)	−0.141	(−0.524, 0.243)	0.728	0.468
24 h ARV of DBP	Sex	−0.902	(−1.773, −0.031)	−2.055	0.043*
	Age	−0.146	(−0.434, 0.141)	−1.011	0.314
	BMI	0.123	(0.013, 0.233)	2.209	0.029*
	Nap duration (hours/day)	0.674	(0.173, 1.175)	2.668	0.009*
	Nighttime sleep duration (hours/day)	−0.183	(−0.436, 0.071)	−1.429	0.156

Sex: Male = 1, Female = 2. BMI, body mass index; DBP, diastolic blood pressure; SBP, systolic blood pressure; ARV, average real variability.

* *P *< 0.05.

## Discussion

In the current study we found that in university students insufficient nighttime sleep and late bedtime were quite common. Longer nap duration was correlated with increased BPV, including daytime CV of DBP, nighttime CV of SBP, 24 h SD of DBP, daytime SD of DBP, nighttime SD of SBP, 24 h-WCV of DBP, 24 h-WSD of DBP, 24 h ARV of SBP and 24 h ARV of DBP. Especially for young adults with nap duration >60 min, 24 h WCV of DBP, daytime CV of DBP, and daytime SD of DBP were significantly higher than those without daytime napping. In addition, with Multiple regression analysis we found that nap duration was an independent predictor for nighttime CV of SBP, daytime CV of DBP, 24 h WCV of DBP, 24 ARV of SBP and 24 h ARV of DBP.

### Prevalence of insufficient nighttime sleep and late bedtime

In this study, we found that the average nighttime sleep duration of college students was 7.52 h higher than the 7.08 h reported in a meta-analysis on the sleep of Chinese college students (9). The average nighttime sleep duration was within the recommended standard of 7**–**9 h. Yet, notably the percentage of participants with nighttime sleep duration ≤6 h was 8.57% and 48.57% for ≤7 h. Among university students, insufficient nighttime sleep has been shown to be detrimental to health (22, 23). Adequate sleep is essential to rejuvenate students each day and help them with learning and memory processing (24). Previous studies have reported that poor sleep quality was associated with poor academic performance (25). Sleep deprivation was identified as a risk factor for poor mental health, even suicidal ideation (26). Meanwhile, sleep deprivation was associated with higher levels of IL-1β, TNF-α, and IL-10 (27). In addition, our study showed that university students generally go to bed at 12:00 PM and later, with 72.12% of students going to bed at 12:00 PM and later. Previous studies have suggested that later bedtime (midnight or later) was related to adverse health outcomes (28). The SBP and the prevalence of diabetes was significantly increased with the delay of bedtime, after 12pm in large community studies (29, 30). Another large-scale association study from several countries found that late bedtime was associated with higher risk of general obesity (aOR = 1.20, 95% CI, 1.12–1.29) and abdominal obesity (aOR = 1.20, 95% CI, 1.12–1.28) compared to bedtime between 8:00 PM and 10:00 PM (31).

### Napping duration and BPV

Daytime napping is a common lifestyle in China (32). However, there have been inconsistent findings about association between napping and health (33). In some cross-sectional studies, it was reported that participants with daytime napping were more likely to develop obesity and diabetes than those without napping (34, 35). Particularly, when combined with short sleep duration, daytime napping increased the risk of type 2 diabetes even more (36). Napping also negatively affected renal health. The risk of microalbuminuria for participants with napping 0–1 h/day, 1–1.5 h/day and >1.5 h/day is 1.552, 1.301 and 1.567-fold compared with those without napping, respectively (37). However, other studies have also reported that short daytime napping were also considered to be beneficial for physical health, in reducing the risk of cognitive decline in older adults (38). A systematic review suggested that napping improved cognitive and physical performance and reduces fatigue in athletes (39). There are also conflicting results regarding the effects of daytime naps on cardiovascular diseases (40). Cao et al. showed that a much higher incidence of hypertension in those who took longer naps compared with those without napping (41). In contrast, a cohort study in China showed that only prolonged daytime naps (≥30 min) increased the risk of cardiovascular events by 22%, and short daytime naps (<30 min) did not increase the risk (42). Differently, another study showed that daytime naps >1 h reduced the risk of hypertension with an adjusted OR of 0.70 (95% CI, 0.51–0.97), and the protective effect of longer daytime naps was still found in the presence of adequate nighttime sleep (43).

In our study, 61.9% of the university students reported participating in daytime napping. We did not find an association between abnormal BP and nap duration (Supplementary Table S1). It is probably the participants in our study are young and in a physical healthy condition. But we found a positive correlation between nap duration and BPV among university students. There are several explanations or potential mechanisms for the findings. Firstly, daytime napping may be associated with inefficient sleep (high level of sleep fragmentation) (44). It has also been shown that inefficient sleep makes BPV increase (17). Secondly, evidence has also linked prolonged daytime napping to inadequate nighttime sleep duration, induced strong inhibition of *γ*-aminobutyric acid receptors in the paraventricular nucleus of the hypothalamus, which could over-activate the sympathetic nervous system, leading to abnormal fluctuations in blood pressure and increased BPV (45–47). Thirdly, previous studies have also found that prolonged napping was associated with higher melatonin (48). The blood pressure pattern during daytime napping is similar to that of nighttime sleep, with a rapid increase in blood pressure after daytime napping (49, 50). Therefore, prolonged daytime napping may alter circadian rhythms, resulting in abnormal fluctuations in blood pressure. Our results revealed that there was a significant difference between the nap duration >60 min group and the no napping (0 min) group, and 24 h WCV of DBP, daytime CV of DBP and daytime SD of DBP were higher in the nap duration >60 min group than the no napping group (*P *< 0.05). Thus far, the majority of studies on sleep and BPV have focused on patients with obstructive sleep apnea and little evidence is available with respect to the relationship between napping and BPV among the general population. Further investigations demonstrating relationships between nap duration and BPV are warranted, especially larger prospective studies.

BPV can be an important predictor of the progression and severity of cardiac and vascular damage, and cardiovascular events and mortality, especially in high-risk cardiovascular populations (51). Increased BPV can induce chronic myocardial inflammation, which exacerbates cardiac hypertrophy and myocardial fibrosis, leading to systolic dysfunction in the hypertensive heart. This may be related to mineralocorticoid receptor systems and activation of the local angiotensin II (52). In women with pre-eclampsia, BPV was observed to be associated with right ventricular strain (53). In patients with type 2 diabetes, E-selectin, an endothelial-specific molecule involved in vascular inflammation and cardiac metabolism, was positively associated with 24 h diastolic BPV(*r* = 0.238) and daytime diastolic BPV (*r* = 0.258) (54). Furthermore, recent studies have proposed that people with high short-term BPV are at high risk for hypertension and should be closely monitored (55). Therefore, it is also necessary to explore the factors influencing BPV. However, there is no generally accepted gold standard index for blood pressure variability until now. In our study, we chose SD, CV and ARV as our BPV indicators. SD is the most commonly used indicator to assess blood pressure variability, which is able to reflect the dispersion of original BP readings (56). The disadvantage is that it depends on the average level of BP and is susceptible to short-term blood pressure fluctuations (51). However, CV is not affected by the mean level of blood pressure than SD and is suitable for comparison with different mean values (56). When assessing 24 h blood pressure variability, the traditionally calculated 24 h SD and CV would be affected by circadian blood pressure variability, so the weighted 24 h SD and CV are considered better indicators (57). Furthermore, ARV was proposed as a more reliable indicator for assessing BP variability, which was calculated as the mean of absolute differences over 24 h between consecutive BP measurements (51). Not only is it not affected by the mean blood pressure level, but it also represents time series variability (58). To eliminate the effect of the mean on the results, we selected the CV and ARV as the dependent variable for multiple linear regression analysis. With gender, age, BMI, bedtime, napping duration, and nighttime sleep time as independent variables, our study found that daytime napping was independently associated with nighttime CV of SBP, daytime CV of DBP, 24h WCV of DBP,24 h ARV of SBP, 24 ARV of DBP. Notably, in the regression model of 24 ARV of DBP, we found that males with a high BMI and long nap had higher ARV. For diastolic or systolic variability, it is inconclusive which is more valuable for predicting poor health outcomes. Systolic variability has received more attention than diastolic variability in studies. Higher systolic variability has been associated with increased risk of all-cause mortality, dementia, coronary heart diseases, stroke, and renal diseases (59, 60). In studies of patients receiving intravenous thrombolysis (IVT) for acute ischemic stroke, higher 72 h BPV between prior IVT to 72 h after IVT had an increased risk of stroke outcome within 3 months, SBP (OR = 5.298, 95% CI, 1.339–10.968) and DBP (OR = 6.397, 95% CI, 1.576–25.958) (61). However, it has been recommended that DBP may predict cardiovascular diseases risk in young adults to a greater extent than SBP (62). Previous studies on daytime napping and blood pressure have mostly focused on elderly populations, and few studies have been reported on younger people. Overall, we obtained more positive results related to diastolic BPV (including daytime CV of DBP, daytime SD of DBP, etc.), especially when comparing different napping time groups. Diastolic variability relevance among youth remains to be further demonstrated.

### Limitations

Our findings help to elucidate the relationship between nap duration and BPV indicators, which will facilitate further in-depth studies. The present study also has some limitations. Firstly, ambulatory blood pressure measurement data were difficult to obtain, so the present study was conducted using a relatively small sample. Secondly, in the present study the sleep data is self-reported, there could be some recall bias. Future studies are recommended to assess napping characteristics more accurately by wearing a wrist actigraphy for multiple consecutive days. Also, our study did not take certain important factors into account, such as dietary information and physical activity that are related to sleep and BP. Future related studies should take these factors into consideration. Finally, this study is an observational study and can only provide correlations between sleep characteristics and BPV and cannot prove the causality.

## Conclusions

Short sleep duration and late bedtime are quite common among university students. Nap duration is independently associated with BPV among university students. Especially, those with daytime napping >60 min had a significantly higher BPV than those without daytime napping.

## Data Availability

The raw data supporting the conclusions of this article will be made available by the authors, without undue reservation.
