# Supplementary material for: Association between napping and 24-hour blood pressure variability among university students: A pilot study
Source: Front Pediatr. 2023 Mar 2;11:1062300. doi: 10.3389/fped.2023.1062300 (PMC10018217; doi:10.3389/fped.2023.1062300)
Supplement: Supplementary file 1 [file Table1.pdf]

Table S1. Logistics regression analysis of abnormal blood pressure based 24h ABPM

Independent Variables	β	SE	OR	95%CI	*P*
Sex	-0.802	0.533	0.448	(-0.158, 1.274)	0.132
age	-0.068	0.184	1.070	(0.747, 1.533)	0.713
BMI	0.019	0.070	1.019	(0.889, 1.169)	0.783
Nap duration(hours/day)	0.070	0.319	1.072	(0.574, 2.003)	0.827
Nighttime sleep duration(hours/day)	-0.098	0.162	1.103	(0.802, 1.515)	0.547

Sex: Male=1, Female=2. BMI: body mass index. Abnormal blood pressure based 24h ABPM: Normotensive=0, Abnormal blood pressure=1.
